# Histopathological Analogies in Chronic Pulmonary Lesions between Cattle and Humans: Basis for an Alternative Animal Model

**DOI:** 10.1100/2012/647403

**Published:** 2012-05-02

**Authors:** Rafael Ramírez-Romero, Alicia M. Nevárez-Garza, Luis E. Rodríguez-Tovar, Alfredo Wong-González, Rogelio A. Ledezma-Torres, Gustavo Hernández-Vidal

**Affiliations:** Pathobiology Group, Faculty of Veterinary Medicine, Autonomous University of Nuevo León (UANL), Avenida Universidad S/N Ciudad Universitaria, 66451 San Nicolas de los Garza, NL, Mexico

## Abstract

Most of the natural cases of pneumonia in feedlot cattle are characterized by a longer clinical course due to chronic lung lesions. Microscopically, these lesions include interstitial fibroplasia, bronchitis, bronchiectasis, bronchiolitis obliterans, and epithelial metaplasia of the airways. Herein, the aim was to review, under a medical perspective, the pathologic mechanisms operating in these chronic pneumonic lesions in calves. Based on the similarities of these changes to those reported in bronchiolitis obliterans/organising pneumonia (BO/OP) and chronic obstructive pulmonary disease (COPD) in human beings, calves are proposed as an alternative animal model.

## 1. Introduction

Pneumonias represent the most prevalent problem in feedlot cattle systems worldwide. Development of new vaccines and antibiotics do not seem to decrease the losses endorsed [1]. The problem is related to younger and lighter-weight cattle with no preconditioning management entering the feedlot. These animals are very susceptible to stress and thus predisposed to respiratory disease [1]. Comparable situations happen during spread of infectious disease in people undergoing vulnerable conditions [2]. Furthermore, the study of respiratory diseases at a population level in the feedlot could be useful for comparative purposes [2].

Literature related to respiratory disease in feedlot cattle is lavish. However, most papers refer to pathogenesis during the acute phase of the lung lesions of shipping fever-related pneumonias [3, 4]. Conversely, only few studies have recorded the chronic progression of lung lesions after experimental induction of pneumonia in ruminants [5, 6]. It is worth noting that pneumonic fatalities in the feedlots have lengthier clinical courses than previously recognized and that chronic bronchopneumonia is the most frequent lesion encountered [7, 8].

Taking into account that most of the microscopic lesions described in natural and experimental cases of chronic pneumonia in cattle and sheep [5–9] have also been reported in chronic cases of pulmonary disease in humans, particularly as components of the bronchiolitis obliterans/organizing pneumonia (BO/OP) [10–12] and chronic obstructive pulmonary disease (COPD) [13–15], herein we sought to compare the pathological mechanisms operating in these chronic inflammatory lesions in the lung. The intention was to propose the calves as an alternative animal model. The arguments are presented considering the histopathological patterns of bronchiolar lesions in humans [16]. These patterns are (a) primary bronchiolar damage, (b) parenchymal lesions with prominent bronchiolar damage, and (c) bronchiolar involvement as a result of large airway diseases [16].

## 2. Primary Bronchiolar Damage

Viral infections are responsible for the initial bronchiolar injury and could be one of the most important elements in the pathological analogies discussed in this paper. Severe viral infections are common in children, calves, and lambs. For instance, respiratory syncytial virus (RSV), included among the most important viruses in children [17–19], is very important in both calves and lambs [5, 19–21]. Certainly, human RSV is able to induce bronchitis and bronchiolitis in neonatal lambs in a manner highly compatible with natural infections in infants [21]. Lambs and calves have been considered as appropriate models of RSV [2, 5, 19–21].

In a previous report in feedlot cattle, bronchitis and bronchiolitis obliterans, included as component of subacute, chronic, or chronic active (bronchiolar) pneumonias, were mostly associated with bovine respiratory syncytial virus (BRSV) immunoreactivity [7]. On this respect, calves experimentally infected with BRSV showed chronic bronchiolitis, including bronchiolitis obliterans at 90 days after infection with viral antigen still demonstrable in the damaged epithelium [5]. Bronchiolitis obliterans is also a common sequel of severe viral respiratory infections in children, particularly with RSV [17–20]. Persistent infection and reinfections with RSV and BRSV in the lower respiratory tract of children and calves, respectively, are quite possible [18–20]. For this reason, it is not uncommon that cases with chronic pneumonic lesions in calves still show areas of active inflammation indicated by the presence of syncytia. Others have previously demonstrated by immunohistochemistry that either, parainfluenza-3 (PI3) and BRSV, are involved in the bronchiolar lesions with syncytium formation in natural cases of bronchopneumonia in feedlot cattle [7, 8, 22].

Similar to severe cases in young children, infection with BRSV in calves is associated with severe histopathological changes in lower respiratory tract characterized by bronchiolitis and interstitial pneumonia with patchy areas of consolidation, atelectasis, and emphysema; occasionally, there is deposition of hyaline membranes [23]. A prominent neutrophilic exudation is associated with an increase in the levels of interferon gamma (IFN-*γ*), tumor necrosis factor alpha (TNF-*α*), and interleukin-6 (IL-6) [23]. In children, delayed sequelae of RSV infection may derive later in airway hyperreactivity and during adolescence and adulthood in asthma or COPD [23].

## 3. Parenchymal Lesions with Prominent Bronchiolar Damage

In addition to viral infection, bacteria are blamed for the most severe parenchymal damage [1, 3, 4]. Severe injury expands from bronchioles and bronchiole-alveolar junction to alveolar spaces. This acute lesion could easily become chronic [9]. Bacteria involved are commonly *Mannheimia haemolytica*, *Pasteurella multocida*, *Histophilus somni,* and *Arcanobacterium pyogenes*; *Mycoplasma bovis* has become very important recently, particularly in chronic cases [1, 4, 7, 8, 22]. Although severe pneumonias still occur in extreme cases in humans [24], this condition is no longer common [10–12].

**Figure 1:**
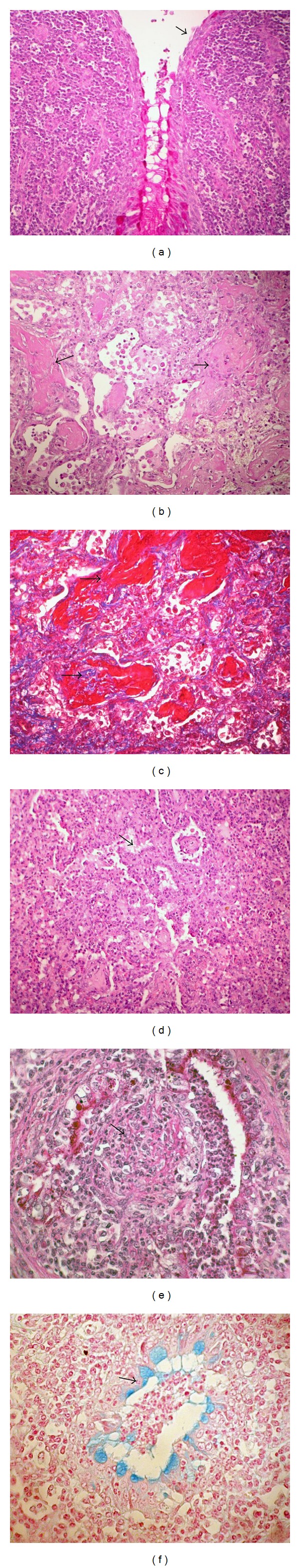
(a) Chronic bronchitis. The lamina propria and submucosa in this large bronchus are completely occupied by lymphocytes and plasma cells. The surface epithelium shows squamous metaplasia (arrows). Desquamated cells are admixed with mucus. PAS stain, 160x. (b) Plugs of fibrin. Fibrin occluding alveolar spaces (arrows) undergoes organization accompanied by active macrophages within alveolar spaces. H&E stain, 160x. (c) Alveolar organisation of exudates with proliferation of collagen within fibrin scaffolds (arrows) as well as in interstitium. Same field as Figure 1(b). Masson's Trichrome stain, 160x. (d) Alveolar epithelialisation. An area almost completely restored with type II pneumocytes proliferation (arrows). H&E stain, 160x. (e) Bronchiolitis obliterans. Bronchiolitis obliterans with various degrees of severity were recorded. In this image, the lesion includes fibroplasia and neovascularisation (arrow). PAS stain, 240x. (f) Goblet cells metaplasia. Epithelial hyperplasia and goblet cell metaplasia (arrows) were recognized in some small bronchioles such as this image with some neutrophils and mucus exudation into the lumen. Alcian blue stain, 240x.

Recent studies in feedlots reported that *M*. *haemolytica* was the most frequently recovered in peracute and acute cases of fatal pneumonia, whereas *M*. *bovis* was related to subacute and chronic cases [7, 8, 22]. Histological observation of the caseonecrotic exudate centred in airway lumen has been consistent with *M*. *bovis* infection [22, 25, 26]. This type of lesion corresponds to severe bronchitis with bronchiectasis, commonly accompanied by bronchiolitis obliterans [26] (Figures 1(a) and 1(e)). Histopathologic resemblance with BO/OP in humans has been mentioned [26].

*Mannheimia haemolytica* also causes chronic damage. It has been demonstrated that both, lipopolysaccharide (LPS) and leukotoxin (LKT), the most important virulence factors in *M*. *haemolytica*, adversely influence the outcome of inflammation in the lung due to an imbalance amongst the enhanced expression of gelatinases (matrix metalloproteinases 2 and 9) and the diminished production of their inhibitors (tissue inhibitors of metalloproteinases) [27]. Similarly, calves with *P*. *multocida* and *Mycoplasma* spp. infection have greater amounts of these matrix metalloproteinases in comparison with those infected with *P*. *multocida* alone, indicating synergism between these organisms [28]. This alteration may contribute to generation of chronic pneumonic lesions which include alveolar fibroplasia, atelectasis and emphysema. These changes are compatible with BO/OP, interstitial pneumonia, and COPD in humans [15, 27–31].

Bronchiolitis obliterans/organizing pneumonia consists of chronic inflammatory and proliferative changes in the pneumonic lungs that arise from an incompletely resolved fibrinous alveolitis. The hallmark is the formation of plugs of fibrinoid material admixed with neutrophils and macrophages occluding alveoli and bronchioles. Subsequently, these scaffolds evolve toward mature connective tissue and promote proliferation of type II pneumocytes (re-epithelialisation) [10–12, 31]. Even though in most cases the lesion occurs accompanied by bronchiolitis obliterans, there are some cases in which the obliterating lesion is not observed, and thus these cases are referred as OP alone [10–12, 31]. This form of pneumonia was originally the common outcome of severe lobar pneumococcal pneumonias in human beings that reached the final stages when antibiotics were still unavailable [10–12]. However, infectious causes of BO/OP in humans are now rare. Nonetheless, a recent case of BO/OP in a boy was associated with a severe immune reaction to *Mycoplasma pneumoniae* infection [24]. The cryptogenic (undefined aetiology or idiopathic presentations of OP) actually the most relevant form of OP (also called primary OP or COP) [10–12, 31] is excluded in this work for comparative purposes.

Typical fibrinous or fibrinosuppurative pneumonias in feedlot cattle are characterised by accumulation of exudates in the alveolar spaces and bronchioles. Organization of this material is one of the most conspicuous images of the pneumonic lesion in calves, which died during severe *M*. *haemolytica* pneumonia [9]. Organising exudates consist of whorls of fibrin admixed with streaming neutrophils (oat cells) inside alveoli and bronchioles [32]. As has been noted, resolution of the lesions in severe cases is incomplete and evolves toward organisation from fibrinous to fibrous plugs covered by type II pneumocytes [26] (Figures 1(a), 1(c), and 1(d)). Experimentally, this change has been seen by 15 days post inoculation and beyond in a model of progression of lung lesions referred in sheep [6]. Therefore, lesions described in BO/OP due to bacterial infections, such as *Streptococcus pneumoniae*, *Mycoplasma pneumoniae*, *Legionella pneumophila*, and *Pseudomonas aeruginosa* [9–12], are similar to those observed in chronic cases of exudative pneumonias (fibrinosuppurative bronchopneumonias) in feedlot cattle. The lobar distribution of BO/OP [10–12] is also in accordance with the pneumonic lesions found in shipping fever [9].

It was postulated that development of BO/OP lesions induced in mice is the result of the severity of initial lung injury inciting an augmented expression of proinflammatory and profibrotic cytokines [33]. This condition is likely to occur in severe cases of shipping fever pneumonia, considering the concomitant role of *M*. *bovis* and continuous exposure to environmental pollutants in the feedlot, which contribute to/or sustain the inflammatory response with progression to fibroplasia [1, 22, 34, 35].

It is worth to mention that recently the chronic lung lesions recognized in pigs naturally infected with porcine circovirus 2 (disease named chronic postweaning multisystemic wasting syndrome [PMWS]) showed similarities with BO/OP [36].

## 4. Bronchiolar Involvement as a Result of Large Airway Disease

Arising from virus-induced bronchiolar insult, air pollution and lipopolysaccharide (LPS) may also instigate the severity and permanence of chronic inflammation with or without bacterial superinfection. For instance, higher morbidity and more severe cases of RSV occur in infants living in zones with high levels of air pollution [18–20]. Correspondingly, the highest morbidity and mortality related to respiratory viral infections occur in younger calves entering the feedlot pen [1].

Respiratory syncytial virus has been linked to cases of severe exacerbation of COPD requiring hospitalisation [20, 37]. Interestingly, RSV infection induces an overexpression of the Toll-like receptor 4 (TLR-4) in lung epithelial cells [18–20]. Toll-like receptor 4 is the signal receptor for LPS, and therefore lung tissue become more susceptible to an exacerbated inflammatory response mediated by LPS through the TNF-*α* pathway [18–20].

Several conditions in feedlots could favour the maintenance of factors that contribute to or sustain the inflammatory response in the lung such as feedlot dust. Feedlot dust contains sufficient amounts of LPS to induce lung lesions and a systemic response in sheep [34, 35]. The presence of proinflammatory stimuli, such as exposure to airborne LPS, is the most likely influence that impedes healing in pneumonic cattle in feedlots [34, 35]. Correspondingly, feedlot workers are more susceptible to severe manifestations of respiratory disease and feedlot dust induces a proinflammatory cytokine profile in human bronchial epithelial cells *in vitro *[38].

Continuous exposure to LPS produces inflammatory changes in the mouse lung comparable to COPD in humans [39]. Similarly, chronic tobacco exposure induces epithelial squamous metaplasia in proximal airways in rats [40]. In cattle, subacute to chronic lesions of suppurative bronchopneumonia are characterized by bronchitis, bronchiolitis, pleural and parenchymal fibroplasia, atelectasis, and emphysema [9]. These histological changes are also compatible with COPD. However, in cattle, squamous metaplasia in large airways is occasionally seen in severe cases with chronic progression (Figure 1(a)). Instead, chronic bronchitis with goblet cells hyperplasia (Figure 1(a)) as well as goblet cell metaplasia in bronchioles is more common [9, 26] (Figure 1(f)). All of these changes are considered examples of adaptive response to persistent insult to the airways and highly representative of COPD [9, 13–15, 40].

Chronic obstructive pulmonary disease is a respiratory syndrome of multiple etiologies that provokes dyspnoea due to obstructive lesions in the intrapulmonary airways. Chronic bronchitis, chronic bronchiolitis, bronchiolitis obliterans, bronchiectasis, and emphysema associated with neutrophil infiltration, macrophages, lymphocytes, and mast cells are always present in severe cases [13–15]. Causes of COPD in humans are diverse and include respiratory tract infections, air pollution, and smoking [13–15, 39, 40]. For the present discussion, we considered the infectious causes and the effects of air pollution in establishing pathological analogies with cattle.

 It has been proposed that COPD is the result of inflammation/restoration events occurring in an unfavourable inflammatory condition leading to incomplete healing and abnormal deposition of extracellular matrix [13–15, 29, 30]. Bronchiolitis obliterans is a common feature in COPD [13–16]. It is also a characteristic of chronic cases of suppurative and fibrinosuppurative pneumonia in feedlot cattle [7–9, 26]. Experimentally, bronchiolitis obliterans has also been observed after development of chronic lung damage induced by *M*. *haemolytica* in sheep [6]. In these cases, persistent lung damage could be the result of a redundant activation of the TNF-*α* pathway [41]. It is most likely that, when viral infection initiates damage and chronic airway inflammation remains, fibrotic changes prevail in the airways and lung parenchyma [14, 15, 29, 30, 39, 42, 43].

## 5. Pathogenesis Outline

**Figure 2:**
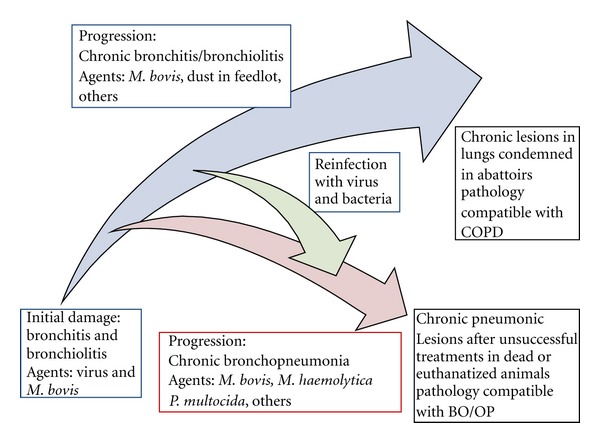
Proposed pathogenesis during progression of chronic lung damage in feedlot cattle and its relationship with BO/OP and COPD. Viral infection provides the original insult. *Mycoplasma bovis* has a prominent role in the progression of chronic lung damage. Treatment with partial recovery may have a progression until the end of the fattening process, leaving sequels that cause condemnations at slaughterhouse. These lesions may resemble partly COPD. Conversely, severe fibrinosuppurative pneumonia with chronic course due to unsuccessful treatment may cause death or terminal lesions. These lesions may resemble in part BO/OP. An overlap between these patterns may occur.

It has been thought that, despite their peculiarities, COPD and BO/OP have in common to be chronic lung pathologies with sequential changes [12]. Undoubtedly, sequential changes are also inherent in the pathological images of chronic pneumonic lesions in feedlot cattle. A proposed pathogenesis of chronic respiratory disease in cattle related to BO/OP and COPD is depicted (Figure 2). In this simplified scheme, original insult provided from viral infection and *M*. *bovis* occurs without major consequences and most of the calves recover. Subsequently, the higher risk for respiratory disease begins at entering to the feedlot system between 8 to 12 months age. At this point, many animals become affected by respiratory disease, some of them do not recover and turn into chronics or die in feedlot, but most of them return to productivity with pulmonary sequelae remaining in a few. In the first case, most of the animals show pulmonary lesions comparable with BO/OP. Conversely, few of the animals clinically normal that reach the productive standards may have lung lesions condemned at the abattoir compatible with COPD.

## 6. Conclusion

An ideal match of natural respiratory disease in animals and humans to date still remains to be impossible. For instance, employing laboratory animals such as mice resulted advantageous. Alternatives for strain homologies, repeatability, gene targeting, and availability of a wide array of reagents and molecular tools became mice the most popular species as animal model [23]. However, between mice and humans there are substantial differences in pulmonary structure and maturity [23]. Furthermore, most models in mice have negligible histological changes in lung [33, 39]. Conversely, in cattle, lung structure and maturity are highly compatible with humans [23, 44]. Additionally, age presentation and pathological evolution of respiratory infections are well matched to the most severe cases of respiratory disease in man [23]. Unfortunately, cattle have reduced collateral ventilation (lobules completely separated by connective tissue and scarcity of pores of Kohn), gravitational influence favours accumulation of exudates in cranial-ventral lobes, and pulmonary intravascular macrophages are abundant [9]. In addition, multiple aetiologies occur during evolution of pulmonary lesions which is extremely rare in humans [23]. Nonetheless, in spite of obvious disadvantages, alternative nonlaboratory animal models such as cattle, could offer benefits in studying respiratory disease [2, 19, 20, 44].

Most of the studies of respiratory disease in cattle have been oriented and interpreted in the context of animal science and veterinary medicine rather to a medical perspective [23]. Considering that models of chronic pulmonary inflammation during growth could elucidate the influence of early inflammatory injury and persistent proinflammatory stimuli on lung remodelling and maturity [43], calves could provide a natural model for this intricate scenario.
